# Supplementary figures and images for: A surface lipoprotein on Pasteurella multocida binds complement factor I to promote immune evasion
Source: PLoS Pathog. 2025 May 6;21(5):e1012686. doi: 10.1371/journal.ppat.1012686 (PMC12080921; doi:10.1371/journal.ppat.1012686)

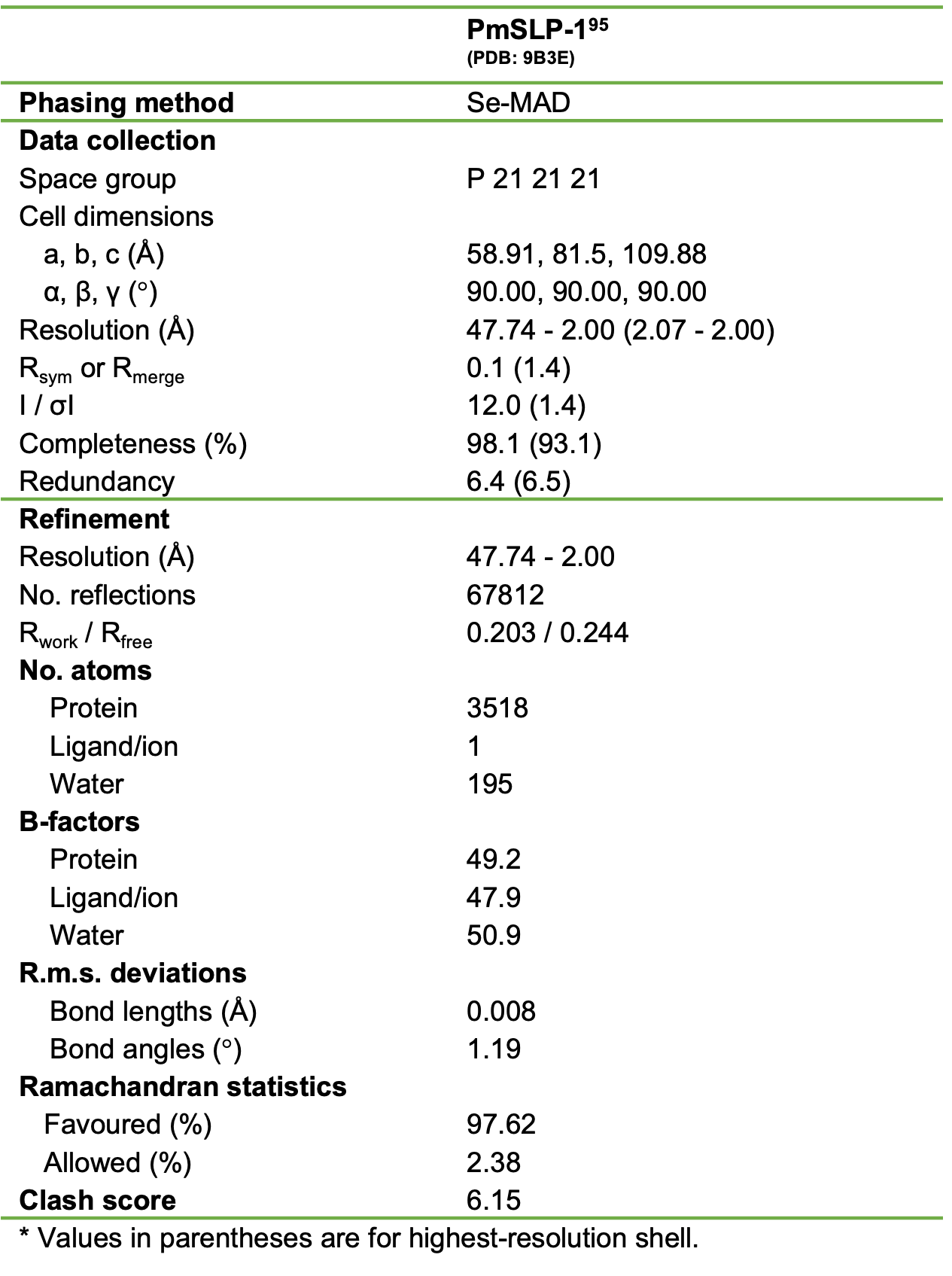

Supplement: S1 Table — (TIF) [file ppat.1012686.s001.tif]

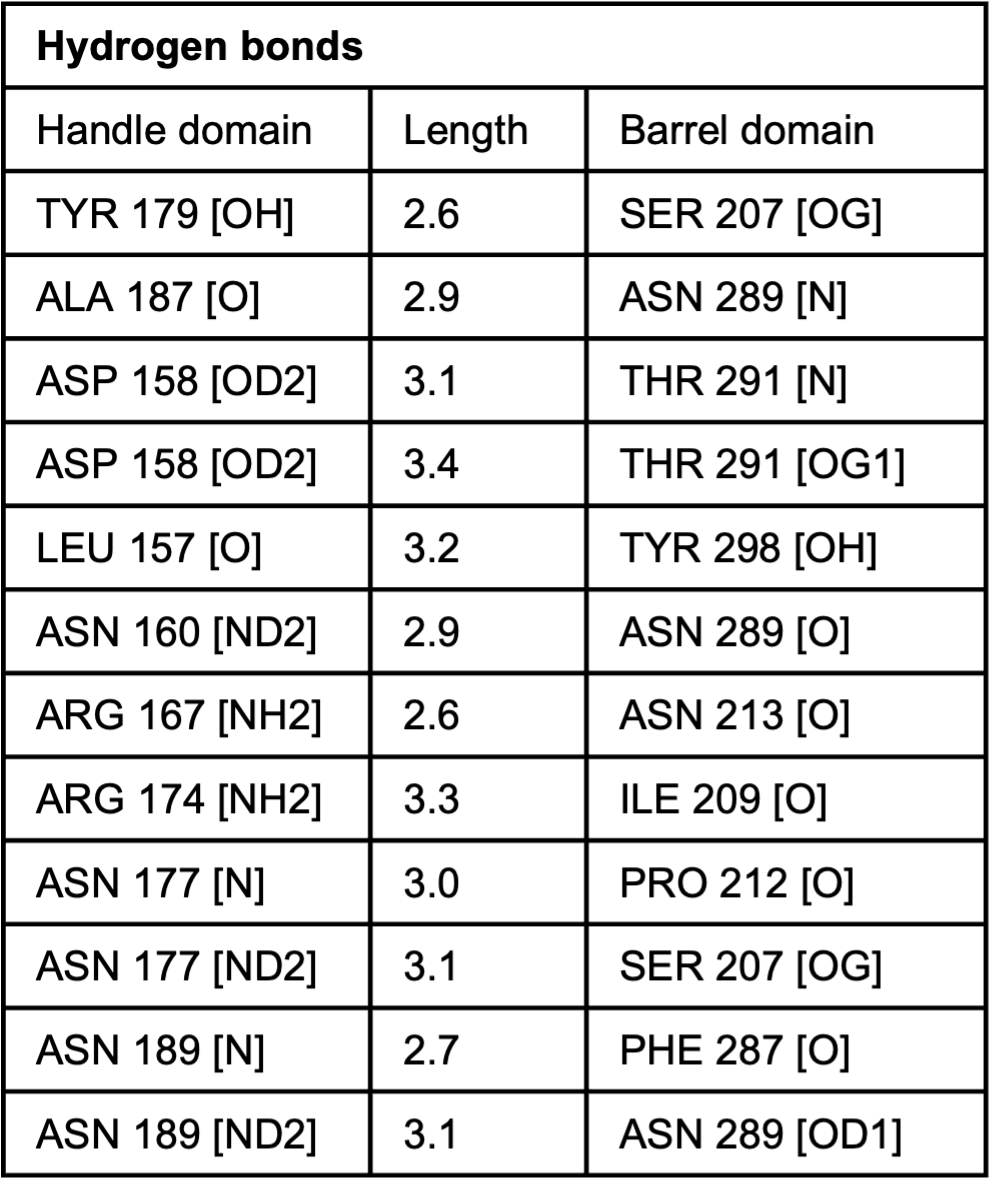

Supplement: S2 Table — The analysis was done with the PISA analysis software. (TIF) [file ppat.1012686.s002.tif]

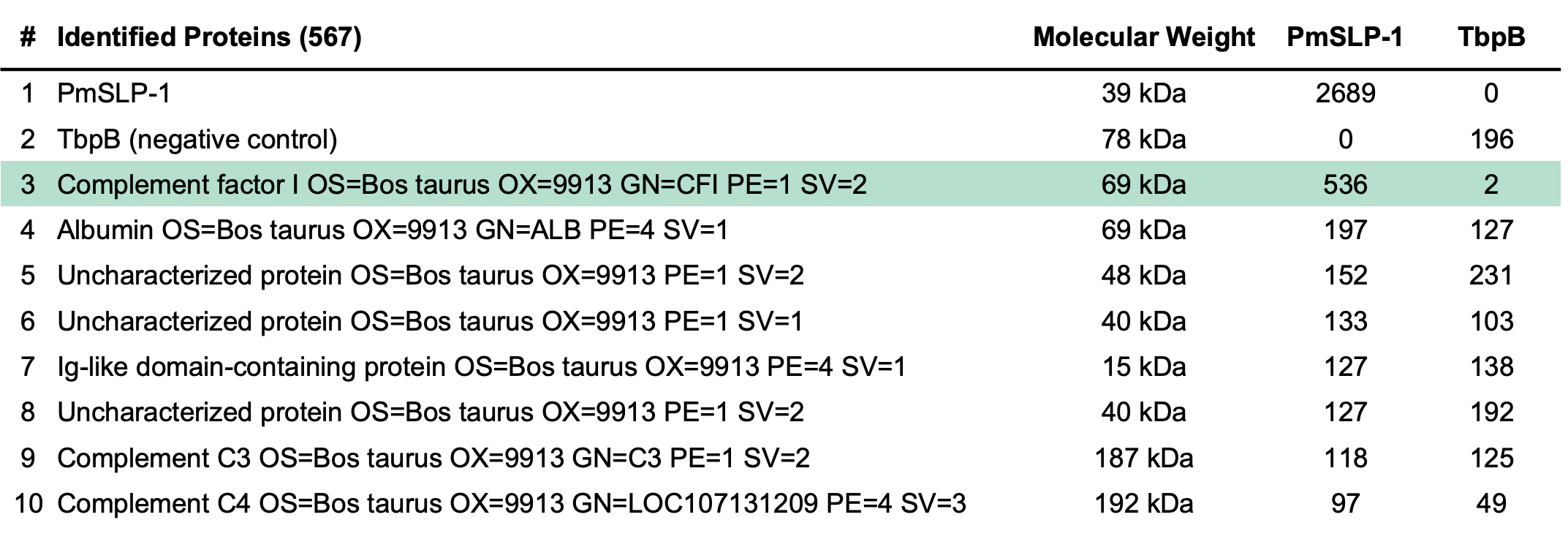

Supplement: S3 Table — Transferrin binding protein B (TbpB) was used as a negative control. The table includes the name of the identified proteins, the molecular weight, and the spectral counts for each identified protein in the target PmSLP-1 sample and in the negative control sample. The hits were sorted in a descending order based on the spectral count in PmSLP-1 sample. Only the top 10 out of 567 hits were shown. The protein with the highest spectral count in the PmSLP-1 sample was highlighted. (TIF) [file ppat.1012686.s003.tif]

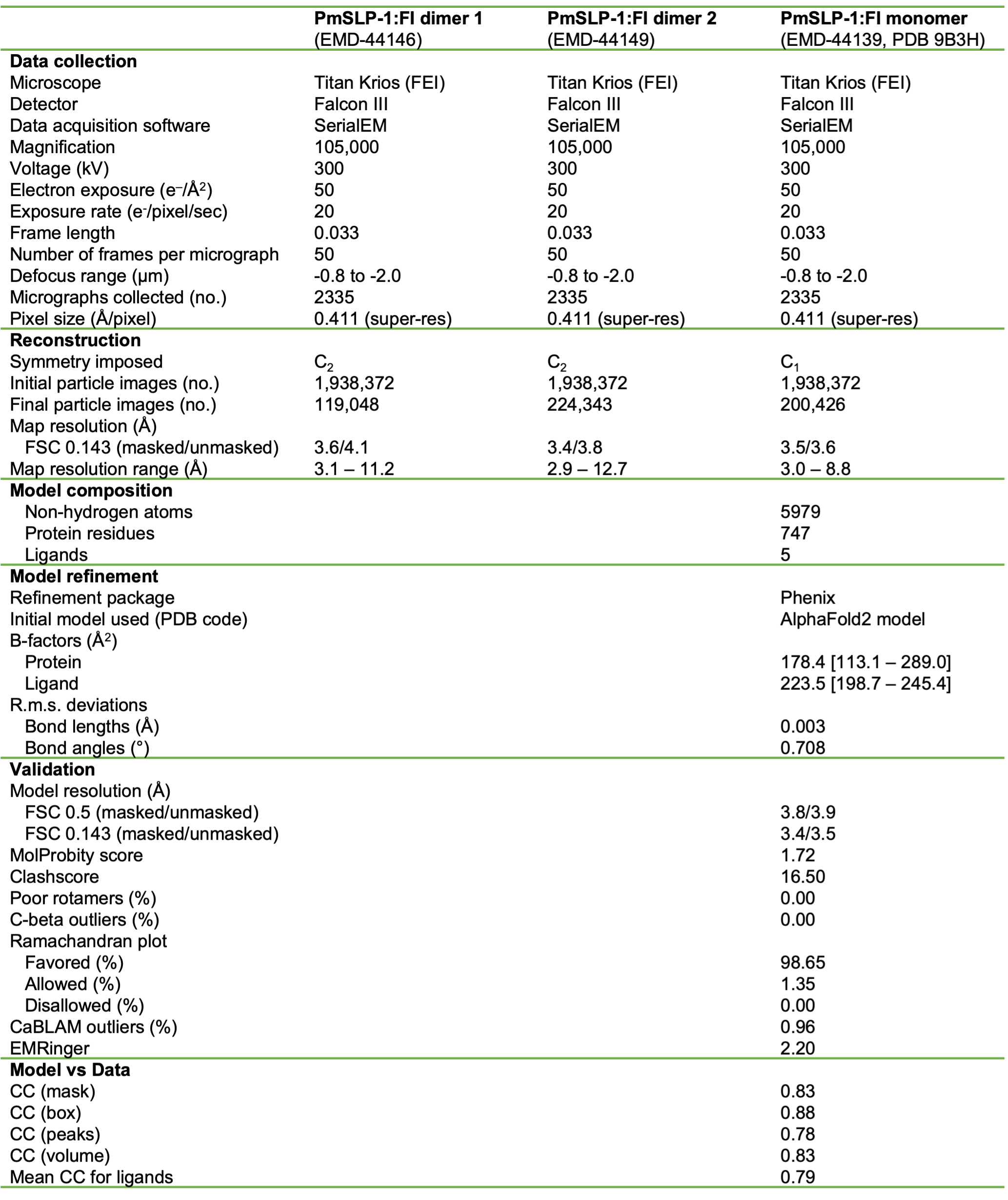

Supplement: S4 Table — (TIF) [file ppat.1012686.s004.tif]

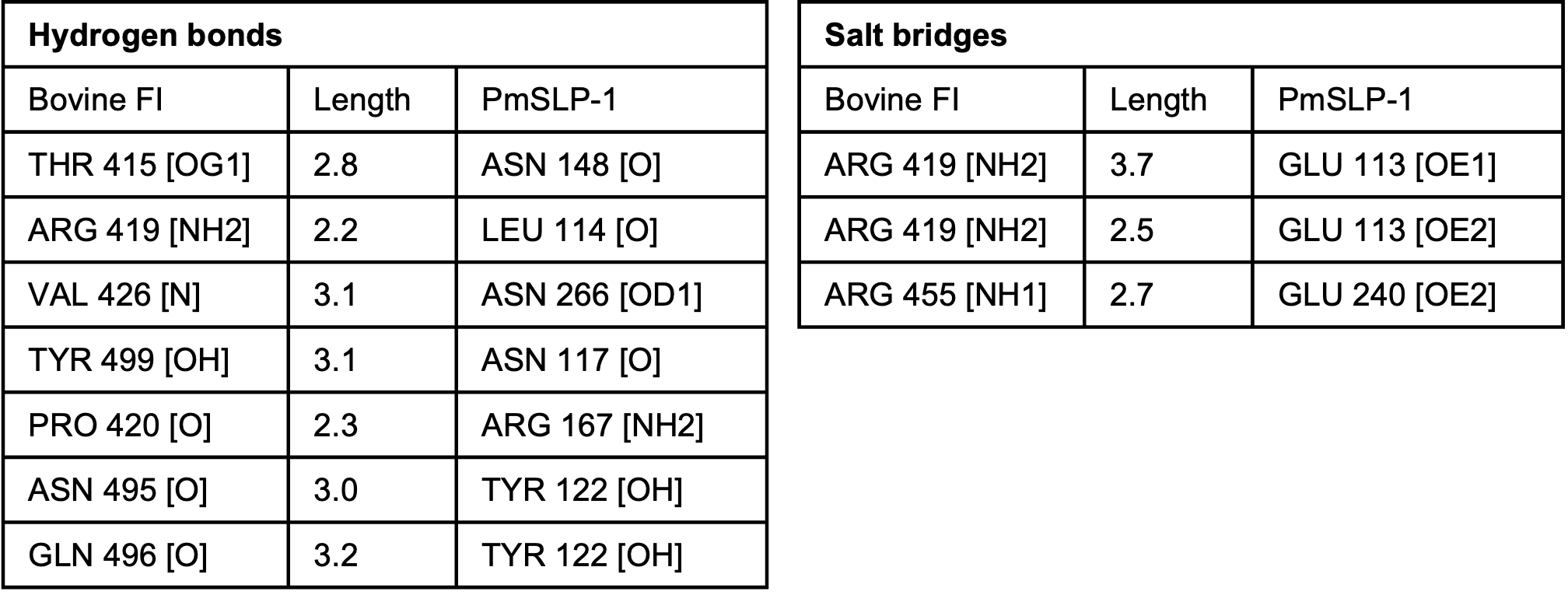

Supplement: S5 Table — The analysis was done with the PISA analysis software. (TIF) [file ppat.1012686.s005.tif]

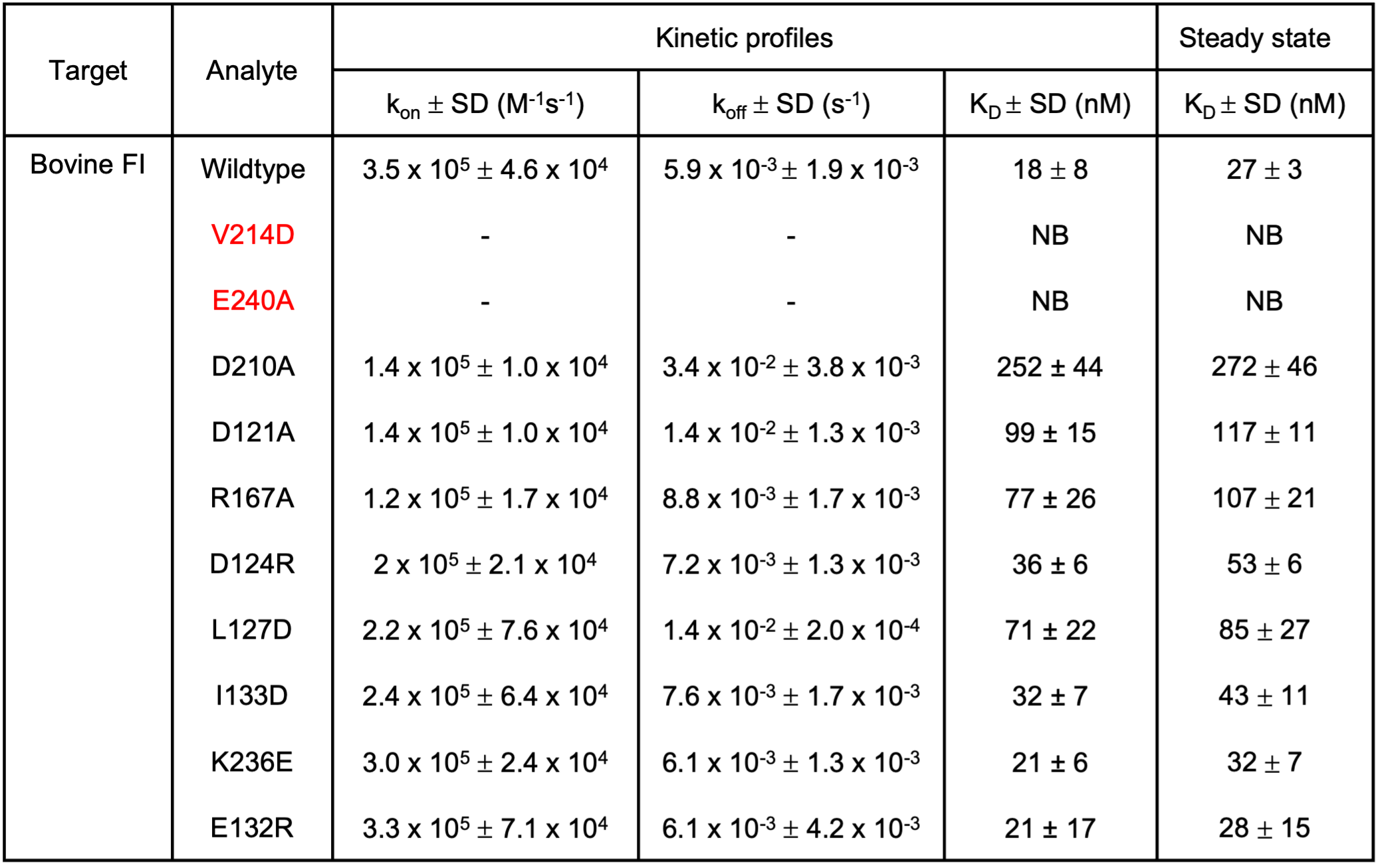

Supplement: S6 Table — Data were analyzed with the Octet Data Analysis software 7.0. For each interaction, a kinetic KD and a steady state KD were obtained. NB indicates no binding. (TIF) [file ppat.1012686.s006.tif]

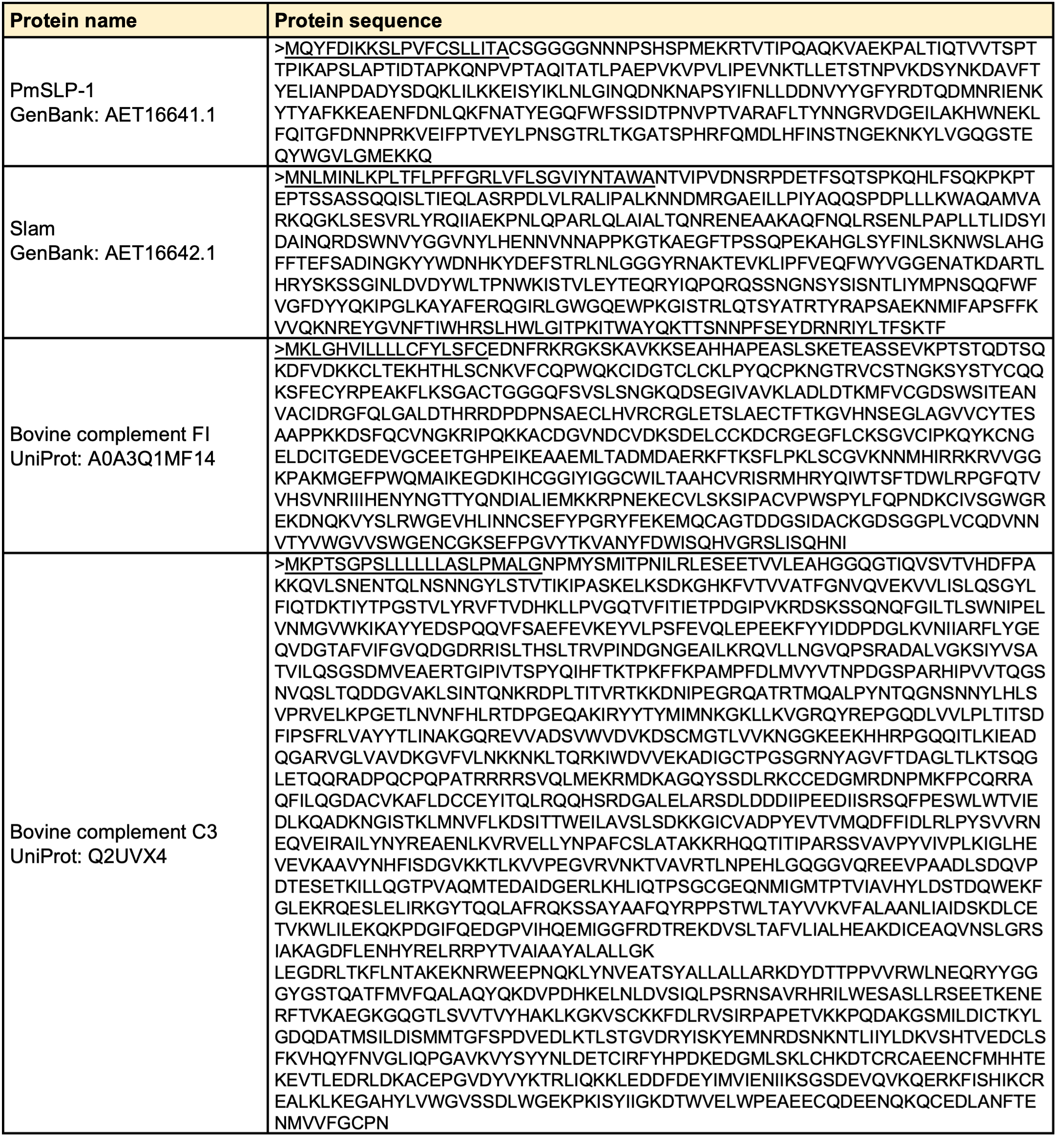

Supplement: S7 Table — (TIF) [file ppat.1012686.s007.tif]

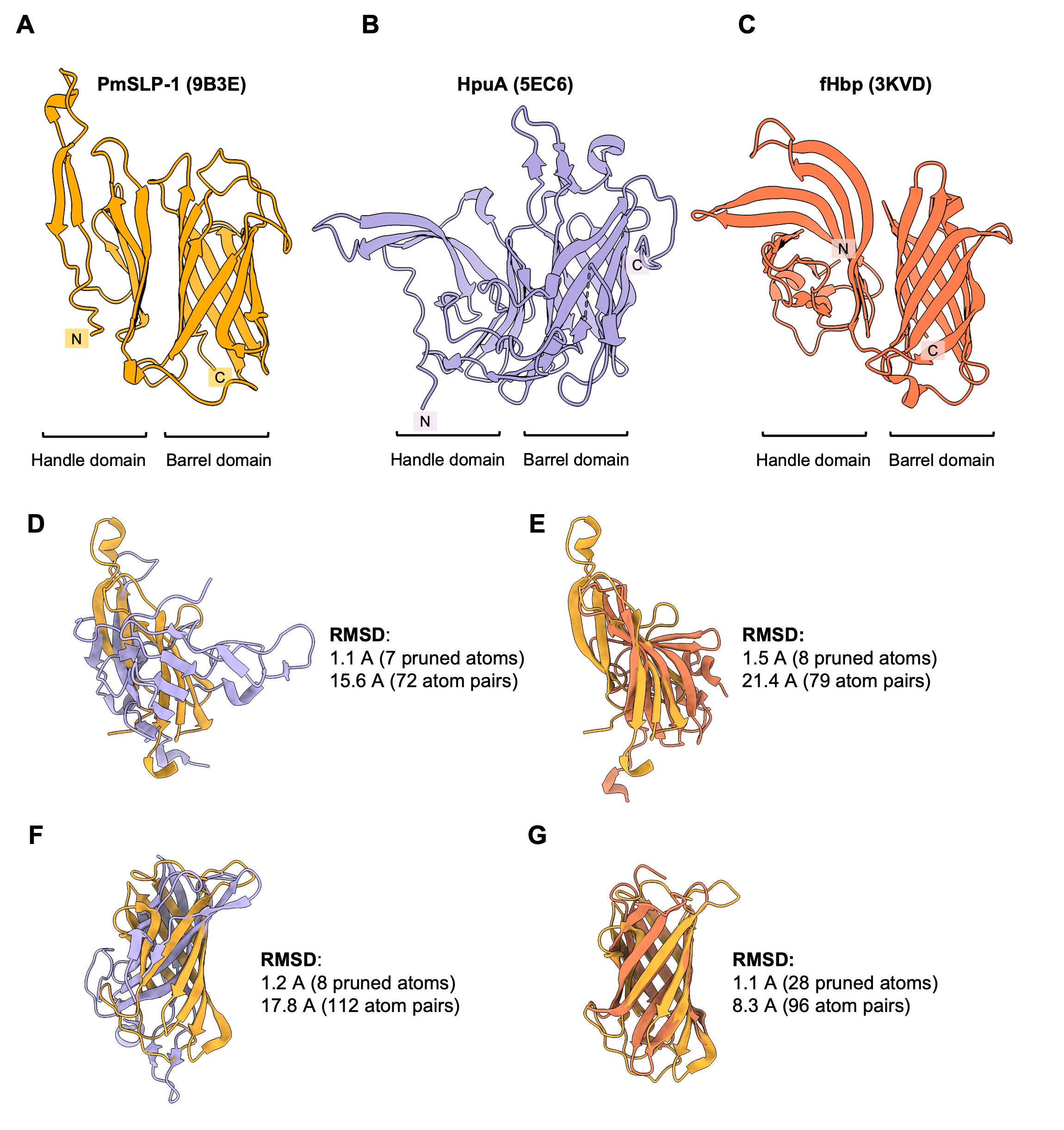

Supplement: S1 Fig — (A-C) High resolution structure of representative Slam-dependent surface lipoproteins: PmSLP-1 from Pasteurella multocida (yellow), hemoglobin receptor from Kingella dentrificans (purple), and factor H binding protein from Neisseria meningitidis (orange). The colour scheme is maintained throughout. The N- and C-termini of each structure are denoted with ‘N’ and ‘C’, respectively. The proteins share similar structural composition, including an 8-stranded β-barrel domain and an N-terminal handle domain made up of 5–6 β strands. Structural similarity between HpuA or fHbp and PmSLP-1 were evaluated in ChimeraX (the alignment is based on secondary structure scoring only) and the reported RMSD values for each domain are indicated above. Structural alignment of the handle domains is shown in (D) and (E). Structural alignment for the barrel domains is shown in (F) and (G). (TIF) [file ppat.1012686.s009.tif]

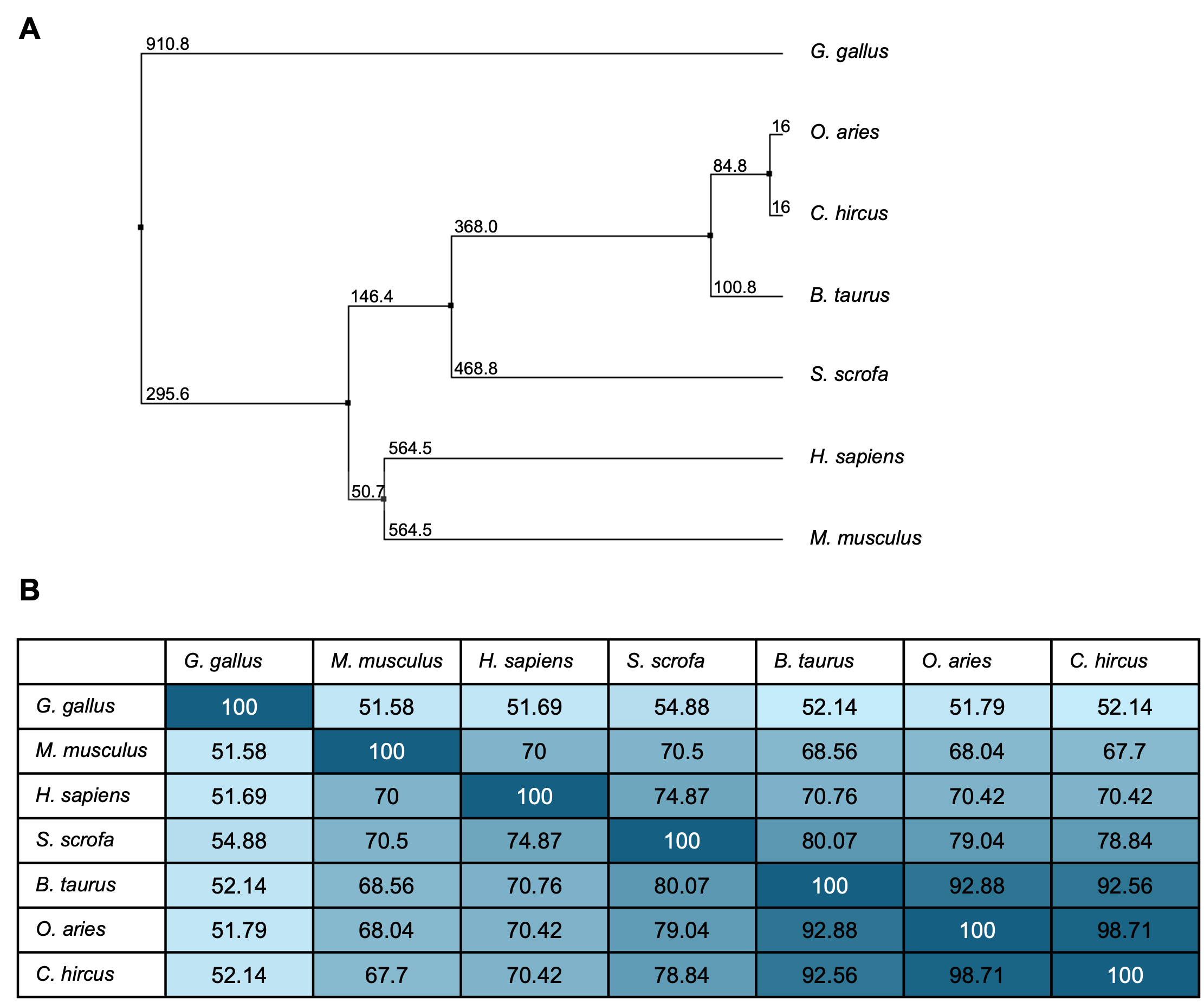

Supplement: S2 Fig — (A) Phylogenetic tree and (B) percent identity matrix of FI homologues from various species. Sequence alignment was performed with ClustalW, and the average distance between each tree node was computed with BLOSUM62. (TIF) [file ppat.1012686.s010.tif]

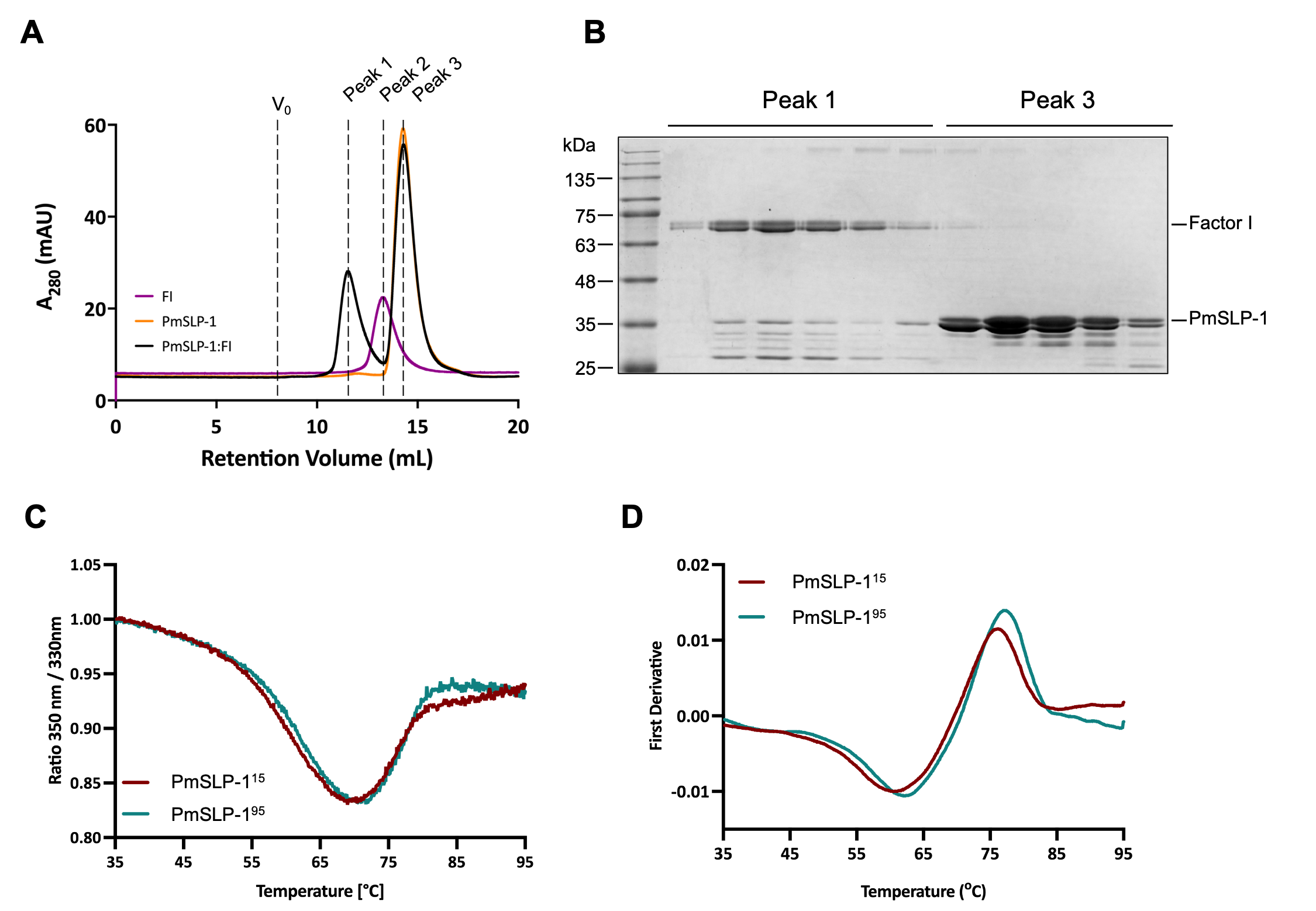

Supplement: S3 Fig — (A) An overlay of SEC elution profiles of purified PmSLP-1 (orange), bovine FI (purple), and a mixture of purified PmSLP-1 and bovine FI (5:1 molar ratio, black). The V0 indicates the void volume. (B) SDS-PAGE analysis of the fractions from peak 1 and 3 of the SEC analysis of PmSLP-1 and bovine FI mixture. (C-D), Thermal stability profiles of PmSLP-115 and PmSLP-195 analyzed by nanoDSF. Changes in the protein intrinsic fluorescence signals under thermal stress (from 35oC to 95oC) are plotted with the raw F350nm:F330nm ratio (C) and with the first derivative of the F350nm:F330nm ratio (D). (TIF) [file ppat.1012686.s011.tif]

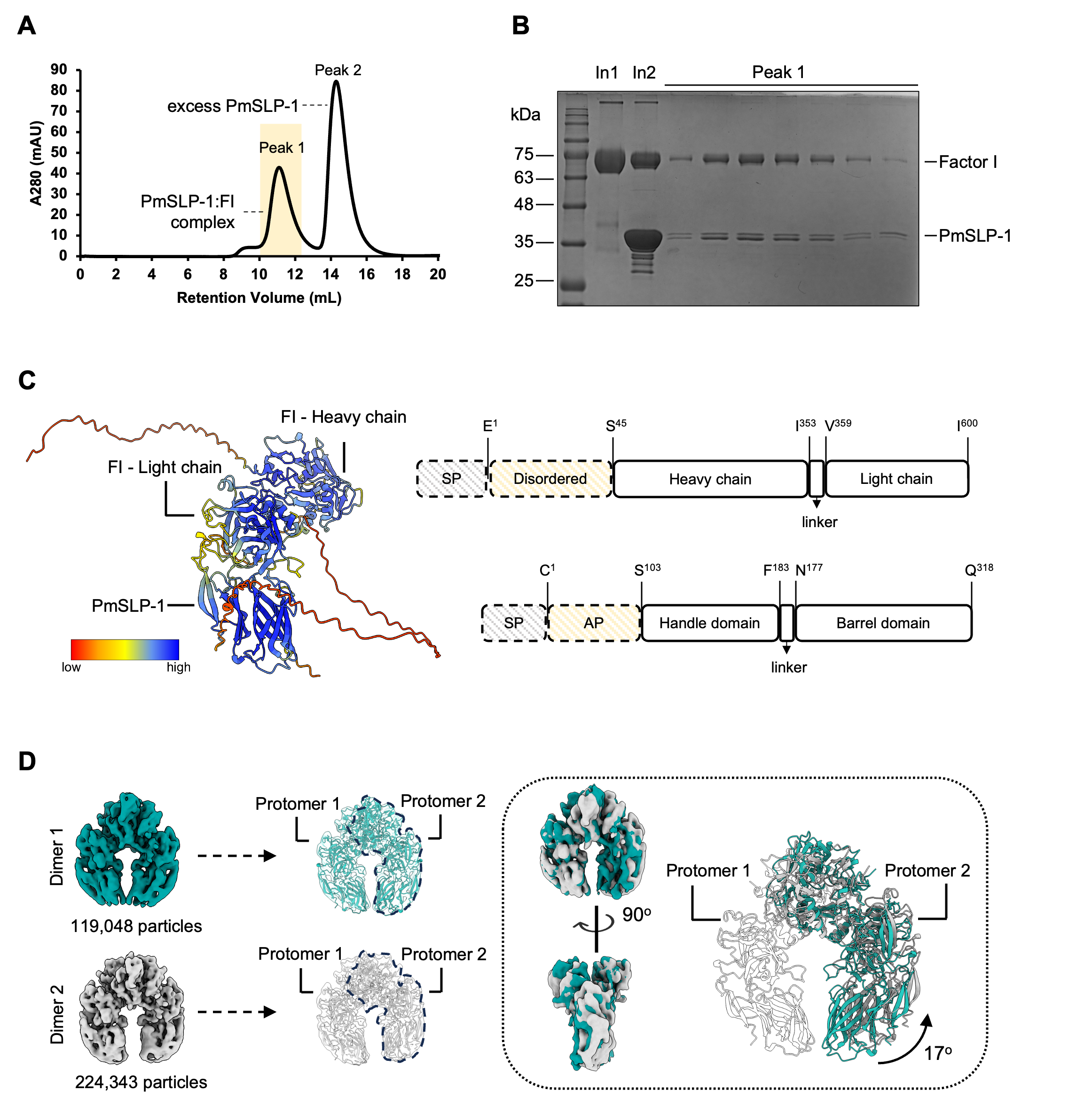

Supplement: S4 Fig — (A) Purified PmSLP-1 and bovine FI were mixed at a 5:1 molar ratio and subjected to SEC analysis. (B) Fractions collected from peak 1 were analyzed on a non-reducing SDS-PAGE gel. Samples from these fractions were pooled, concentrated, and used to prepare Cryo-EM grids. “In1” denotes purified bovine factor I alone, and “In2” contains the input mixture of purified bovine FI and PmSLP-1 prior to gel filtration analysis. (C) AlphaFold2 model of PmSLP-1:FI, coloured by the pLDDT score. The full-length protein sequences (excluding the signal peptide sequence) of the mature proteins were used as inputs in AlphaFold2 (right panel). The N-terminus of both proteins were predicted to be disordered and were removed from the model during the model building process. (D) Cryo-EM 3D classes of the PmSLP-1:FI sample showing a two-fold symmetry indicating dimerization of the protein complex. The AlphaFold2 predicted model were docked into the cryo-EM maps to demonstrate that the PmSLP-1:FI complex dimerization occurs through FI. Overlaying the docked models and aligning them on protomer 1 also revealed a 17o rotation of protomer 2 in dimer 2 (grey) relative to protomer 2 in dimer 1 (teal). (TIF) [file ppat.1012686.s012.tif]

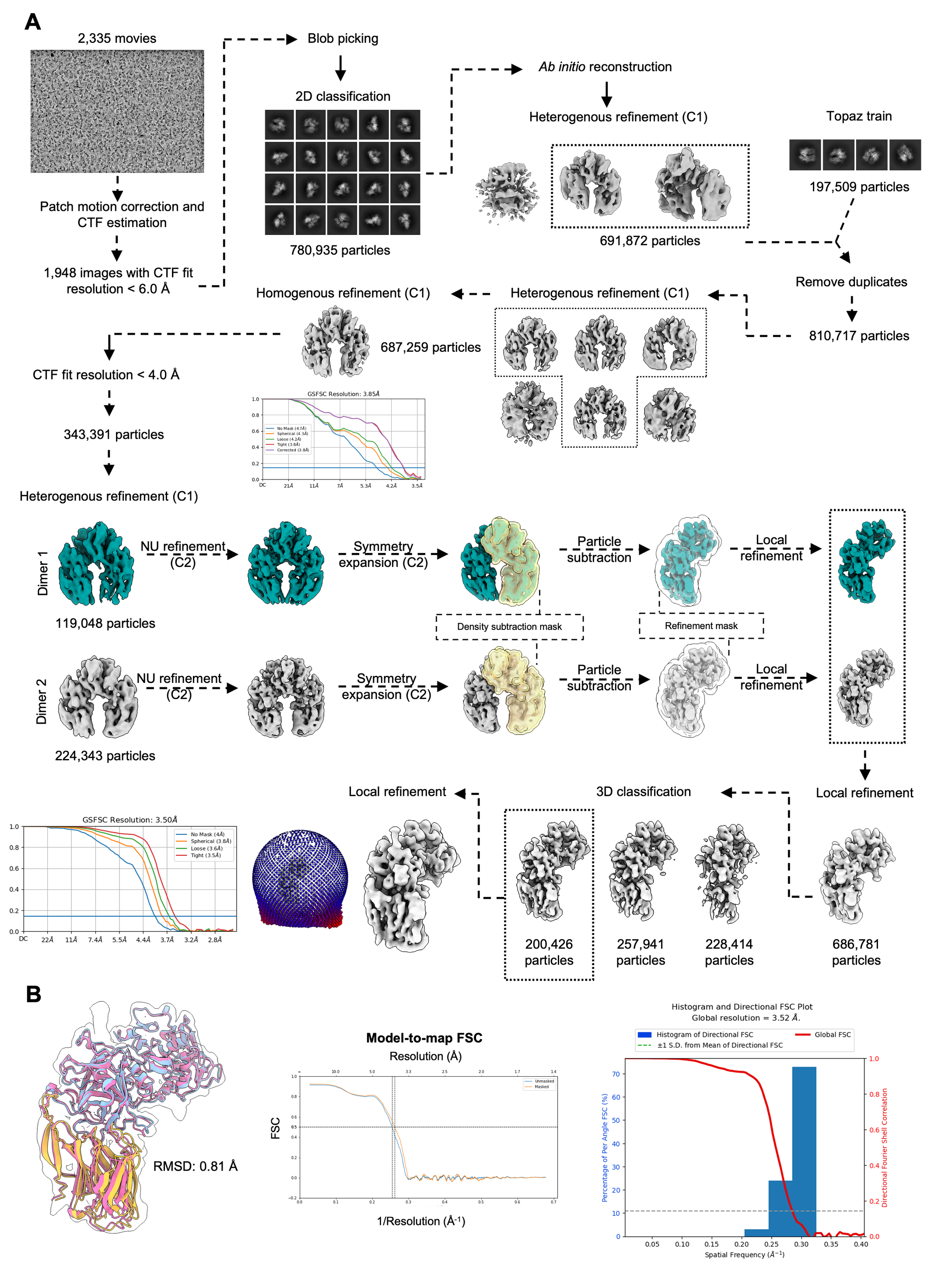

Supplement: S5 Fig — (A) A visual representation of the image processing workflow used to reconstruct the structure of PmSLP-1:FI complex. CryoSPARC was used to obtain the final consensus, local refined maps. (B) The final reconstructed model of PmSLP-1 (yellow) in complex with bovine FI (blue) shows minor differences to the AlphaFold2 predicted model (pink). The model-to-map FSC curve (middle) calculated in Phenix and the 3D FSC plot (right) show how well the model fits in the cryo-EM map and the quality of the consensus map, respectively. (TIF) [file ppat.1012686.s013.tif]

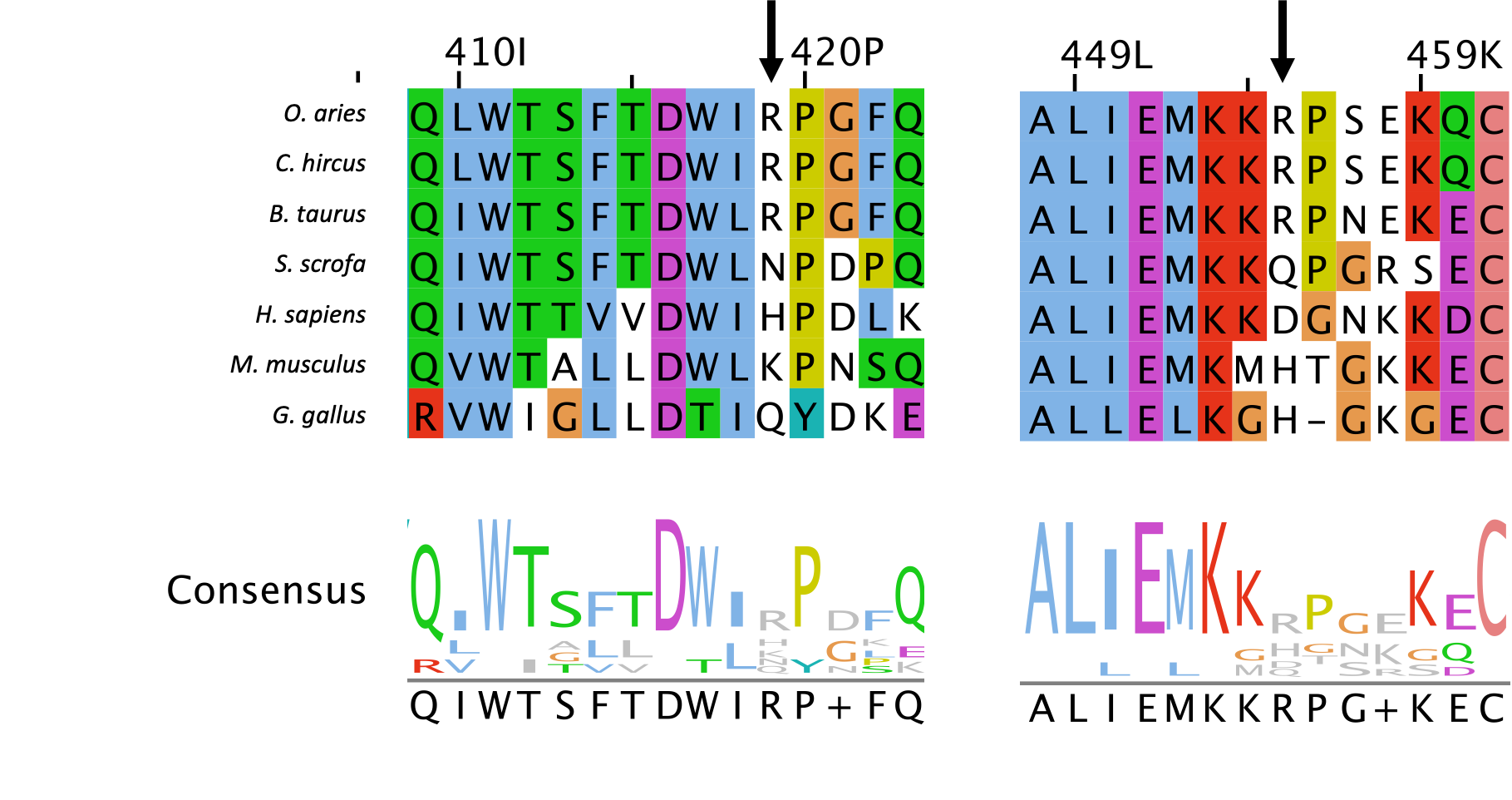

Supplement: S6 Fig — The alignment was performed with ClustalW and colored according to the convention. Bovine FI (bolded) was used as the reference sequence. The arrows highlight the residues in bovine FI that form salt bridges with PmSLP-1. (TIF) [file ppat.1012686.s014.tif]

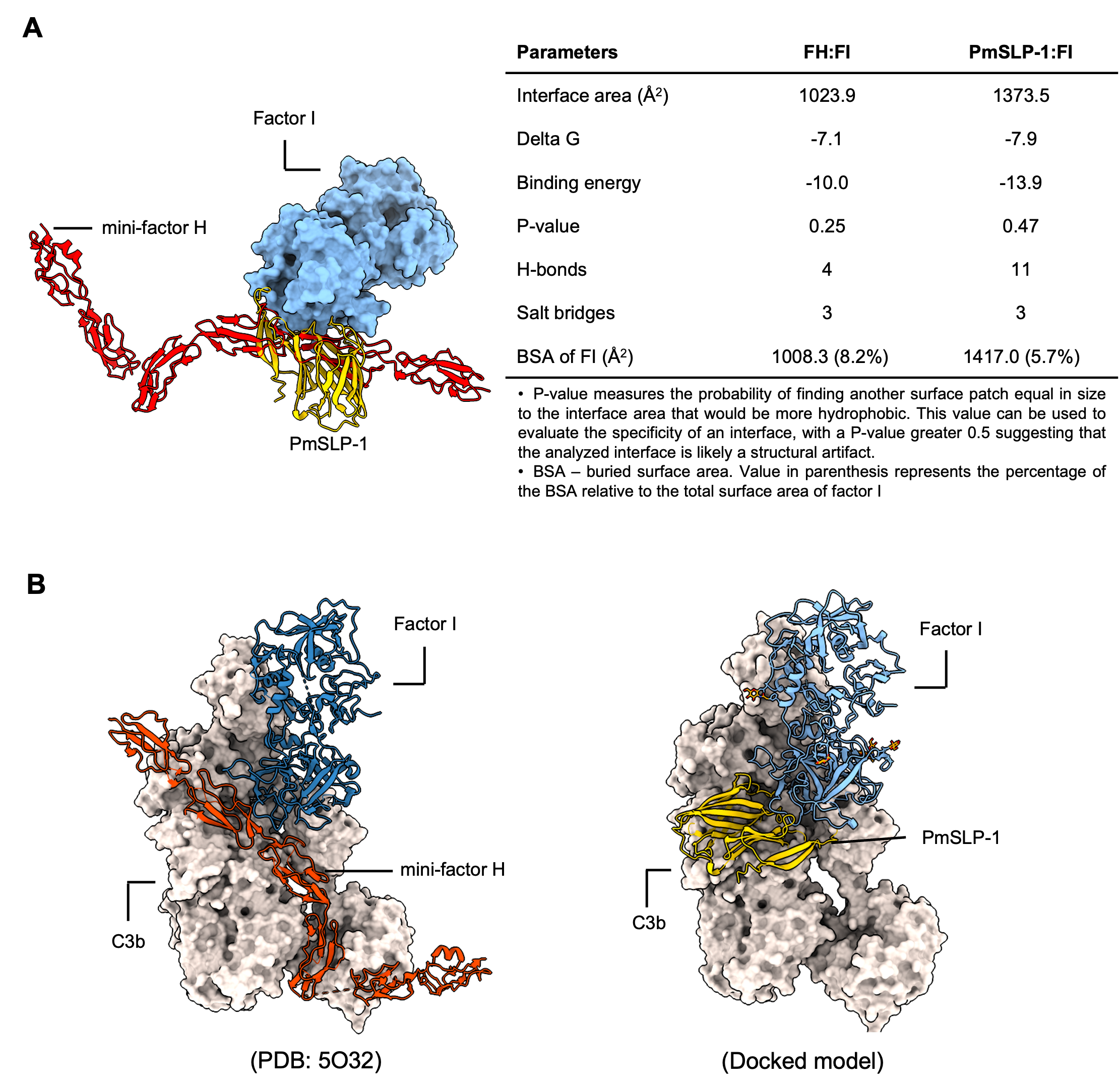

Supplement: S7 Fig — (A) Overlay structures of human mini-FH:FI complex (adapted from PDB: 5O32) and PmSLP-1:FI complex showing that FH and PmSLP-1 share the same binding interface on FI. Protein interfaces were analyzed with the PISA software (as part of the CCP4 package), and the interface parameters for each complex were reported. (B) Crystal structure of human mini-FH:FI:C3b (left, PDB: 5O32) was used to guide the modeling of PmSLP-1:FI:C3b (right). Surface representation of C3b (white) was used for clarity. (TIF) [file ppat.1012686.s015.tif]

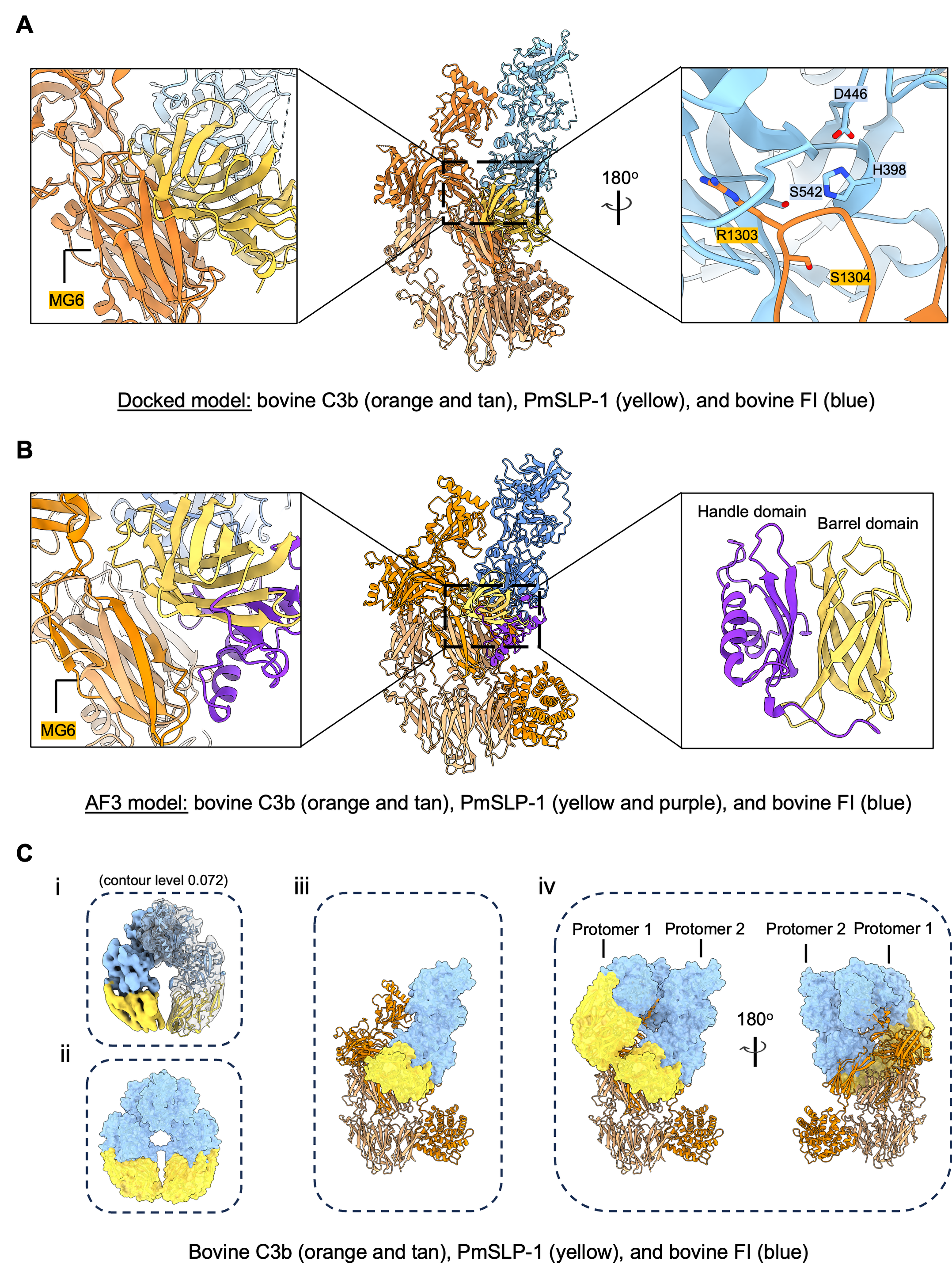

Supplement: S8 Fig — (A) Ternary complex of PmSLP-1:FI:C3b modelled based on the crystal structure of human mini-FH:FI:C3b. Inset shows a detailed view of the clashes between PmSLP-1 and the MG6 domain of C3b (left panel) and the catalytic triad of bovine FI and the first scissile bond on C3b (right panel). (B) AlphaFold3 model of PmSLP-1:FI:C3b. The left inset shows the interface between PmSLP-1 and the MG6 domain of C3b. The right inset shows the AF3 structure of PmSLP-1 alone highlighting the incorrectly predicted handle domain, coloured in purple. (C) A representative cryo-EM map of the PmSLP-1:FI complex dimerizing through the heavy chain of FI (i). Two copies of the solved cryo-EM structure of PmSLP-1:FI monomer were docked into the cryo-EM dimer map, and the docked model is represented as a coloured surface (ii). The AF3 predicted model for the PmSLP-1:FI:C3b complex was combined with the solved cryo-EM structure of PmSLP-1:FI to create a new ternary model. PmSLP-1 and FI are shown as surface representation in yellow and blue, respectively; C3b is shown in cartoon representation (iii). The final panel (iv) shows an overlay of the PmSLP-1:FI dimer (ii) and the ternary complex model (iii) illustrating the clash between C3b on one protomer with FI of the other protomer. (TIF) [file ppat.1012686.s016.tif]

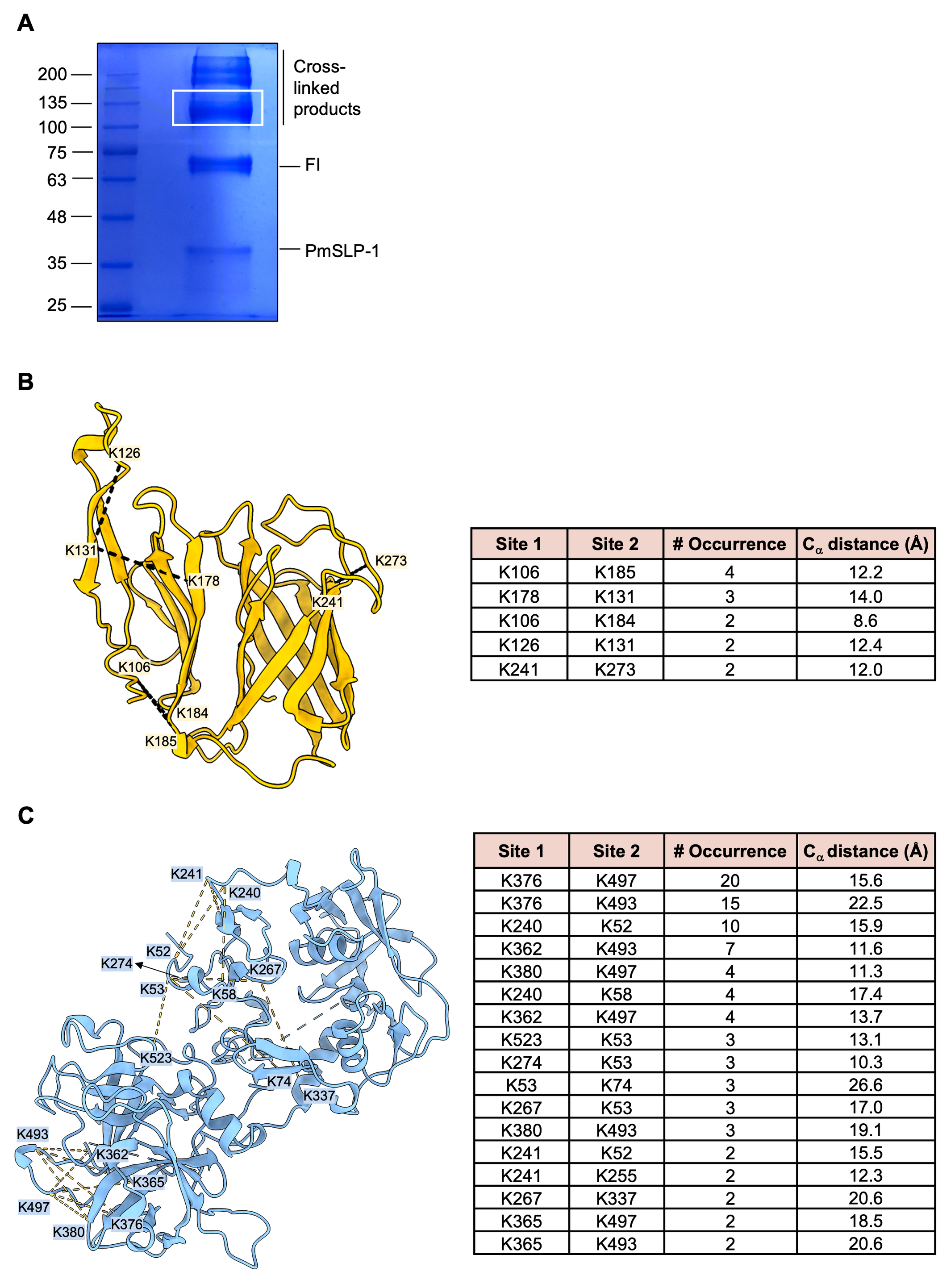

Supplement: S9 Fig — (A) SDS-PAGE analysis of the cross-linked sample. White box indicates the gel band that was excised and subjected to mass spectrometry analysis. (B) Intra-protein crosslinks mapped onto the crystal structure of PmSLP-1. The crosslinked residues, the number of occurrences per crosslink, and the estimated Cα distance are reported. Crosslinked peptides containing residues in the disordered anchoring peptide of PmSLP-1 were excluded. (C) Intra-protein crosslinks mapped onto the solved cryo-EM structure of bovine FI. The crosslinked residues, the number of occurrences per crosslink, and the estimated Cα-Cα distance are reported. (TIF) [file ppat.1012686.s017.tif]

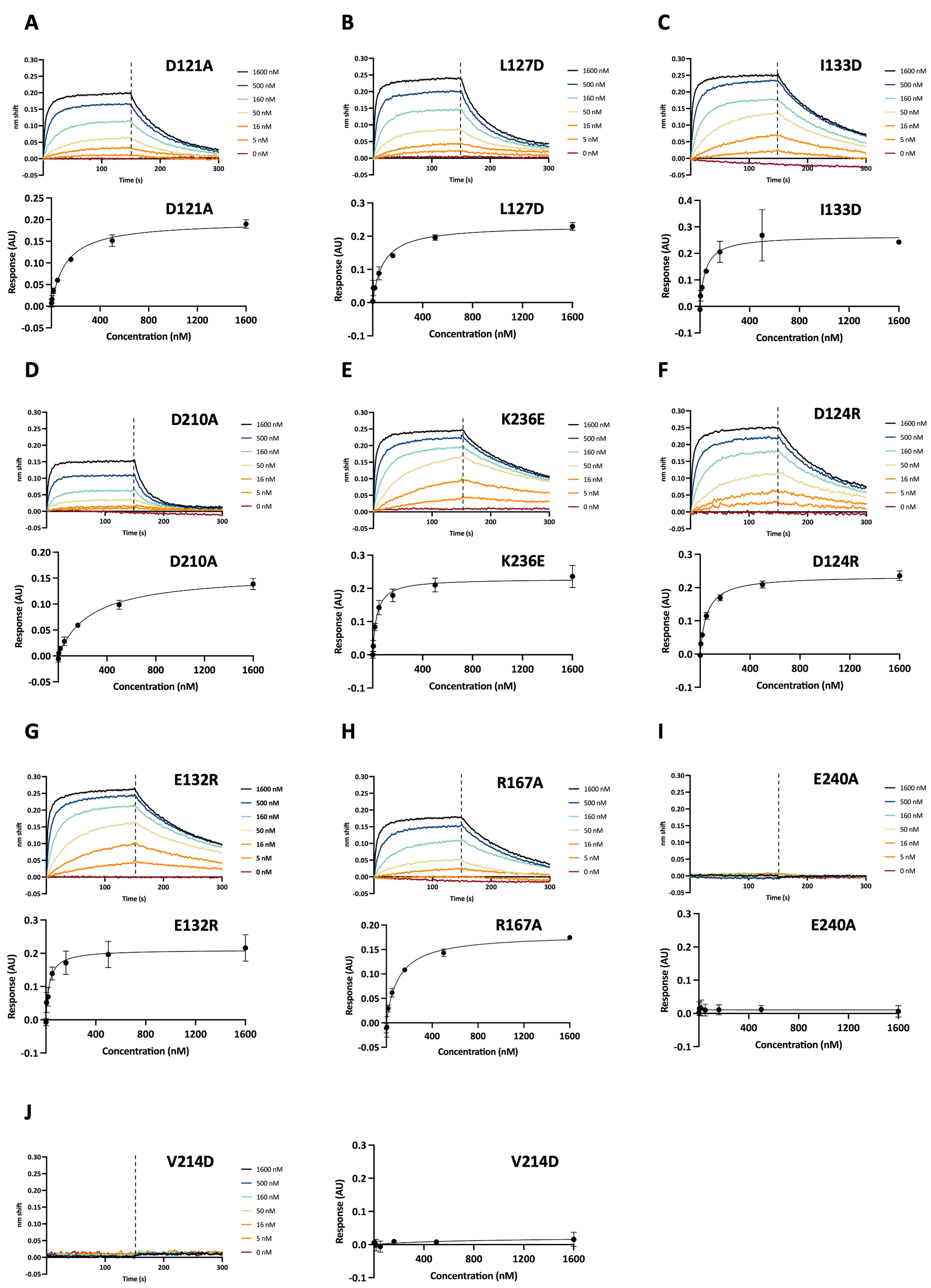

Supplement: S10 Fig — (A-J) BLI sensorgrams and saturation curves showing the binding between bovine factor I and PmSLP-1 mutants. Biotinylated bovine FI was immobilized onto streptavidin sensors, and binding was measured at various concentrations of PmSLP-1. The raw sensorgrams are representative of one replicate for each mutant. The saturation curves are plotted using data from 3 independent replicates. Error bars represent standard deviation. (TIF) [file ppat.1012686.s018.tif]
