# Supplementary material for: A surface lipoprotein on Pasteurella multocida binds complement factor I to promote immune evasion
Source: PLoS Pathog. 2025 May 6;21(5):e1012686. doi: 10.1371/journal.ppat.1012686 (PMC12080921; doi:10.1371/journal.ppat.1012686)
Supplement: Attachment — Submitted filename: Response-to-Reviewers-PLoSPathogens-2025-02-18-TFM.pdf [file ppat.1012686.s020.pdf]

Please find our "Response to Reviewers'" comments in red below.

Reviewers' Comments:

Part I - Summary

Please use this section to discuss strengths/weaknesses of study, novelty/significance, general execution and scholarship.

Reviewer #1: The results presented are impressive and obtained with a large array of complementary techniques and the experimental approach is sound. The mechanistic conclusions are justified considering the data presented, and the discussion covers most required topics. Overall, the manuscript represents an important contribution to the field of complement biology, and especially to our understanding of the molecular mechanisms behind pathogen complement evasion. The manuscript deserves publication after revision that considers the comments below.

Reviewer #2: Summary: This manuscript describes characterization of a novel complement evasion mechanism deployed by the livestock pathogen, Pasteurella multocida. Per the authors' description, P. multocida is a Gram-negative zoonotic pathogen that causes severe diseases associated with high rates of mortality in cattle, including bovine respiratory disease and hemorrhagic septicemia, as well as wound infections in humans. Owing to their recent studies toward P. multocida vaccine development, the authors interest has turned toward an outer membrane protein known as PmSLP-1. A recent paper from the same group (Islam et al, PLoS Pathogens, 2023) has shown that PmSLP-1 is an effective vaccine antigen that provides protective immunity in cattle. However, the function of PmSLP-1 remains unknown. This is the subject of the authors' present investigation.

The authors begin by using a heterologous E. coli expression system to show that PmSLP-1 is exposed on the bacterial surface. This experiment provides some basic architectural information on the PmSLP-1 protein, which is bolstered by solving a high-resolution crystal structure of a large fragment of the PmSLP-1 extracellular region (residues 95-318). Thereafter, the authors "go fishing" for potential PmSLP-1 binding partners in bovine serum, whereby they identify a nanomolar-affinity interaction with complement Factor I (FI). This interaction is mediated by the structured region of PmSLP-1 (i.e. residues 95-318). It also occurs with FI from sheep and goats, which are two other potential hosts of P. multocida. The authors then go on to define the structure of the PmSLP-1/FI complex using cryo-EM methods. They subsequently validate their structural observations by characterizing a relatively large panel of site-directed mutants in PmSLP-1 and by using amine-reactive crosslinking to define contacts between the two binding partners; the results of these two sets of experiments are internally consistent with the PmSLP-1/FI cryo-EM structure. In the last section of the results, the authors show that the PmSLP-1/FI complex alone is sufficient to promote cleavage of C3b and C4b (using human C3b and C4b as surrogates for the bovine protein). They conclude their experiments by demonstrating that heterologous expression of PmSLP-1 in E. coli allows for bacterial growth in diluted, complement-preserved bovine serum, so long as the PmSLP-1 protein is correctly localized to the cell surface. This demonstrates that

recruitment of FI to the bacterial surface by PmSLP-1 provides a measure of protection against complement-mediated killing. Thus, the authors propose that PmSLP-1 be renamed “FI-Binding Protein (fibp)”.

Evaluation: This is a well-conceived and well-executed study that primarily employs a biochemical approach to define a novel complement evasion mechanism. The manuscript was a pleasure to read, and placed the emerging results from the current study within an appropriate context alongside the existing literature in the field. The observations presented should be of interest to those working toward vaccine development against P. multocida and related organisms, but also members of the complement community. The structural data and biochemical data are convincing on their own. However, demonstrating direct recruitment of the FI protease to its substrates (i.e. C3b and C4b) in the absence of known regulatory molecules, such as CR1, FH, and C4BP, is novel to my knowledge and therefore quite exciting.

An additional point that is likely to go unappreciated by many concerns the organization of the FI active site, as described in lines 253-275. FI - like many proteases within the complement system - requires binding to exosites contributed by either a cofactor molecule or regions of the substrate distal to where proteolytic cleavage itself occurs – to assemble an enzymatically-active catalytic triad. The authors’ observations with the covalent inhibitor, PMSF, as well as from their PmSLP-1/FI cryo-EM structure (see Fig. 4f) show that PmSLP-1 binding is sufficient to trigger those changes needed to result in FI enzymatic activity. Thus, PmSLP-1 could be a useful tool for subsequent biochemical studies of FI that may not have been practical otherwise.

Despite my obvious enthusiasm for this manuscript, there are a few small typographical errors that should be corrected prior to acceptance. I have also identified three more substantive concerns that should be addressed. While I’ve listed all of these below, the third point is the most important: an additional well-defined set of experiments is needed to fill the lone gap in this story.

Reviewer #3: Pasteurella multocida is an important pathogen and apart from its zoonotic potential affecting cattle and has a significant economic impact, so understanding of the mechanisms of its pathology is highly relevant and of broad interest.

The present manuscript by Nguyen et al., follows on a pioneering work on the same topic by the same group - (Islam et al., 2023 https://doi.org/10.1371/journal.ppat.1011249), reporting on the isolation of the novel surface lipoprotein PmSLP and its different isoforms.

Here, the study is extended to the structure of the dominant isoform PmSLP-1 both in isolation and in complex with complement factor I, which is identified to be an effector of the PmSLP-1. The latter cryo-EM structure also elucidates the mechanism of FI stimulation by the PmSLP by stabilizing the catalytic domain.

The finding is significant not only as it rationalizes the action of the PmSLP, which appears to be a promising vaccine candidate, but also reports for the first time the direct activation of complement factor I by a bacterial protein.

The protein PmSLP is delivered by the recently characterized Type XI secretion system and is transported across the outer membrane by a dedicated Slam protein. As such this study also provides major leap in understanding of this poorly studied secretion system that is widely spread across the proteobacteria.

The work is well-executed and with its broad, interdisciplinary scope and significant novelty, it merits publication in the PloS Pathogens with minor corrections.

We thank the reviewers for their very thorough and positive reviews of our manuscript. We have put responses to the thoughtful suggestions below.

Part II – Major Issues: Key Experiments Required for Acceptance
Please use this section to detail the key new experiments or modifications of existing experiments that should be absolutely required to validate study conclusions.

Generally, there should be no more than 3 such required experiments or major modifications for a "Major Revision" recommendation. If more than 3 experiments are necessary to validate the study conclusions, then you are encouraged to recommend "Reject".

Reviewer #1: Authors determine the structure of a two-fold symmetric dimer FI:PmSLP-1 complex by cryo-EM. The dimerization appears to be through FI. Discuss whether the dimeric complex is in vivo relevant, and can FI access the C3b substrate in the dimeric state? Is it expected that the dimer form on the surface of Pasteurella multocida? – We don't believe that the FI:FI dimer interface is biologically relevant and may be an artifact of the vitrification process. The effects of FI dimerization on its function and the relevance of this dimerization *in vivo* are discussed at two locations in the manuscript. Please refer to lines 256-265 and 317-326 for more detailed information. While the complex dimerization was an interesting observation, we did not observe heterogenous binding between bovine FI and PmSLP-1 in solution in our BLI experiments (Fig 2C). Additionally, previous studies on the structure of FI in solution indicate that FI remains a monomer even at a concentration that is higher than its serum concentration (Ref 27 and 28 in manuscript). In our studies, the concentration of FI did not exceed the serum concentration, so it's unlikely that the PmSLP-1:FI form an oligomeric complex in solution.

With regards to C3b accessibility in the dimeric state, we docked a model of PmSLP-1:FI bound to C3b into the dimeric map and found significant clashes between the CTC domain of C3b of one protomer with FI of the other protomer (S8 Fig). This suggests that the dimerization of FI would prevent access to C3b and is functionally inefficient.

Multiple panels with detailed views need to be improved considerably, see specific comments. – Noted and modified, please see updated figures.

Reviewer #2: Major Points:

1/ Figures, Fig. 3, 4, Ext4, Ext5, and Ext7: Although I'm not familiar with Journal style guides, the figures mentioned here are problematic because they extend across more than one printed page. I'm sure things will be rearranged and scaled accordingly (if accepted for publication), but it's confusing and inconvenient to have to flip pages to evaluate information contained within a single figure itself. – Noted and modified, please see updated figures.

2/ BLI Studies: The authors present a large number of BLI experiments to determine affinities of wild-type PmSLP-1 and various mutants for bovine FI. While these experiments themselves are not problematic, it seems that the authors chose only to use dose-response analysis to evaluate their data. However, there ought to be kinetic information, such as on-rates and off-rates, obtainable from these experiments. At a minimum, it would be good to show these kinetic rate constants for the wild-type protein. The mutants can be compared to wild-type by dose-response analysis, as is already done, for the sake of simplicity. – We have added the kinetics data (kon, koff, and the kinetic K_D) for the wildtype PmSLP-1 as well as the mutants. We have also modified Fig. 2 to include the raw BLI traces with the fit lines for PmSLP-1^{15} and PmSLP-1^{95}.

3/ PmSLP-1/FI binding to C3b and C4b: The greatest weakness in this study the lack of direct binding data between the PmSLP-1/FI complex and the target molecules, C3b and C4b. To their credit, the authors point this out in the Results (lines 327-330), wherein they suggest that the interaction must be transient as neither C3b nor C4b was observed in their pulldown experiment. While I agree that bands corresponding to either C3b or C4b were not found in the pulldown (see Fig. 2a, b), it's also true that blood/plasma/sera samples are often collected in the presence of agents like EDTA, citrate, etc., that inhibit complement activation; unfortunately, the experimental section doesn't say much about how the serum was obtained, other than the fact that it was taken from healthy donor cattle. Nevertheless, there should be negligible C3b and C4b in the samples used for the pulldown because a specific complement activator was likely not present in the first place. – We have edited the method section to clarify that the pull-down assay was done with commercially purchased bovine serum (line 774). The bovine plasma collected from healthy donors was used only in the bacterial growth assay. With regards to the presence of C3b/C4b in serum for the pull-down assay, we were able to detect the C3 and C4 split products in the same pull-down assay with other PmSLP variants (we have a new manuscript that describes this interaction that will be sent out shortly). We believe this detailed information is beyond the scope of this initial report of PmSLP-1 functioning in immune evasion through FI activation.

Independent of any potential problems with their explanation outlined above or how the serum was obtained, I think the authors should investigate the affinity of the complex they discovered here - as well as that of PmSLP-1 itself - for bovine C3b and C4b. I note that the authors used biotinylated proteins as ligands for their BLI studies. In that regard, there exist numerous protocols for labeling C3b or C4b at the thioester cysteine exposed upon

activation of C3 and C4. Purification of C3 and C4 from serum is straightforward and has been done for decades, and bovine serum is plentiful. So, attempting to address this weakness wouldn't necessarily mean "reinventing the wheel", so-to-speak. On the other hand, addressing this issue will fill the single most obvious gap in the results of this manuscript, which is otherwise very strong.

We agree that the binding of the substrates is a gap in our current manuscript. Herein, we describe how PmSLP-1 binds to FI with high affinity and turns over its substrates (C3b and C4b) very quickly. We tried to detect the interaction between C3b and PmSLP-1 (up to 5 μM) using BLI but did not observe any noticeable binding. However, we were able to trap other homologs of PmSLP (-2, -3, and -4 but not PmSLP-1) in complex with C3b/C4b and FI. We believe that the structures of these PmSLP variants in complex with the substrates and FI, as well as their affinities are beyond the scope of this initial investigation, and the detailed mechanisms of these PmSLP variants will be addressed in our follow-up manuscript.

Reviewer #3: As mentioned above, this is a thorough and well-executed piece of work, which doesn't appear to require any major additional experimental validation to support its central claims. It also has sufficient novelty and impact to justify its appearance in PloS Pathogens.

Part III – Minor Issues: Editorial and Data Presentation Modifications
Please use this section for editorial suggestions as well as relatively minor modifications of existing data that would enhance clarity.

Reviewer #1:
Minor comments

Grammar needs to be checked throughout. In many places "the" is missing. – Thank you. We have double-checked the manuscript and corrected throughout

General note on figures. In many of the close-up views, the perception of depth can be improved by reducing the clip size (distance between the two planes between which things are visible) in the direction perpendicular to the screen. Several such figures would benefit from clip optimization. One example is the close-up view in ext data Fig 5d. – Noted and modified, please see updated figures.

Fig 1C should be improved and expanded considerably. Color the domains differently and present details of the structure. Include a panel illustrating the quality of the electron density, e.g. from the domain interface (see modified figure 1C). Are the two domains stably associated; what type of interactions are keeping the domains together? The expanded view with the three lysine mutations, why is this important for the reader? – Noted and modified Fig. 1C. The list of hydrogen bonds between the two domains has also been added (see line 152-153 and S2 Table). The expanded view with the 3 lysine mutations has been replaced with the electron density at the domain interface (Fig. 1C).

Ext table 1. Provide statistics for Ramachandran and clash score – Ramachandran statistics and clash score have been added to Table S1.

Line 123. Improve language – Noted and modified, please see line 149-152.

Line 157. SEC is 2C not 2D – Noted and corrected, please see line 220. The SEC profile has been moved to supplementary information (S3 Fig).

Ext Fig 3C-D. Please superimpose the fitted curves on the measured association and dissociation curves. Is a 1:1 binding model assumed? – Noted and modified, please see updated figures 2C and 2D.

Line 188. How close was the AF (alphafold) predicted model to the experimental structure? – We have provided the RMSD measurement to compare the AF model with our experimental structure. Please see line 269-272 and S5B Fig.

Line 190. The interface is given as 1374 Å2 (is it an area, so Å2, not Å). Since this means that you suggest the uncertainty is 1 Å2 then why use the word approximately? And this is not buried surface area instead of interface area? Is the area from PISA analysis – Noted and corrected the unit for the interface area. The word "approximately" has been removed (line 279). The value provided is interface area obtained by PISA analysis.

Line 193-194. Please describe the hydrophobic interactions, provide a figure presenting these hydrophobic interactions. – Noted, please see line 280-286 and Fig 3B.

196-197. It seems like Ext fig 2 should be moved, it is much more meaningful after having presented the structure. – Noted and modified, please see S6 Fig.

Line 203. It should be stressed that PmSLP-1 and FH do not share structural homology. It is not correct to state that they interact in the same manner, instead they recognize the same patch on FI, which is something different. Could the FH-PmSLP-1 overlap be quantitated in some way? – We edited the text to emphasize that PmSLP-1 and FH do not share structural homology but bind to the same region on FI (line 292-293). We also added to our PISA analysis table the buried surface area of FI in both complexes as well as the percentage of the BSA relative to the total surface of FI to quantitate the overlap between PmSLP-1 and FH (S7 Fig).

Line 207. The overlap w MG6 in C3b is interesting. Any thoughts on how this could be overcome or whether perhaps pMSLP-1 does not need to form specific interactions with C3b? Could there be conformational changes in C3b or PmSLP-1 that could allow formation of ternary complex. Have authors attempted to measure formation of a ternary complex experimentally? - In this paper, we do not have any experimental data to address the potential conformational change of C3b. However, we have a follow up paper which includes another PmSLP, FI, and C3b in complex in which we will discuss in more details the movement of the MG6 domain. Unfortunately, our attempt at forming a ternary complex with PmSLP-1 on BLI was unsuccessful. We have instead provided additional

information (AlphaFold3 modelling) to describe how PmSLP-1 could form a ternary complex with FI and C3b (see line 313-326).

Line 213. Specify which complex. – Noted and corrected, please see line 348.

Line 236. This is important and impressive data, but the location of the mapped residues and their interaction partners in FI across the intermolecular interface deserves to be presented much better. The overall cartoon-based location in fig 3e is not that informative in this respect. – Noted and modified, please see the updated Fig. 4.

Line 330. Bovine C3 is easy to purify and bovine C3b can be obtained with mild trypsin treatment. It would strengthen the manuscript considerably if the BLI experiment included also bovine C3b. One could also immobilize bovine C3b through thioester Cys-Maleimide-biotin to sensor and attempt a build-up with the preassembled PmSLP-1:FI complex. This is a standard approach for human C3b interaction measurements. The fact that the complex is not isolated in pulldown does not exclude an interaction with Kd in the low micromolar range. – Unfortunately, our attempt at measuring the ternary complex with PmSLP-1, FI, and C3b using BLI was unsuccessful. We tried multiple approaches with PmSLP-1/ FI but could not detect the interaction with C3b even at μM concentrations – we suspect this weak interaction may be a result of the mechanism of PmSLP-1. We do have homologs of PmSLP that do complex with FI and C3b/C4b, which are the focus of a follow-up manuscript.

Line 346. The FI interaction with the CTC may not be mandatory, e.g. see PMID: 32938727. Differences between the rotation of CTC may be influenced by crystal packing. – We appreciate the information. We have elaborated the discussion regarding the interaction between PmSLP-1, FI, and C3b (line 298-320 and 528-542).

Line 351. FI SP is not highly disordered. Regions may be dynamic in free FI compared to FH:C3b:FI complex. Disordered has a special meaning in structural biology which is different from the manner it is used here. – Noted and reworded, please see line 536.

Fig 5. There is no need to duplicate for AP and CP/LP, the principle is the same – We respectfully disagree as we want to highlight that in the presence of PmSLP-1, the FI-mediated processing of C3b ends at the formation of iC3b.

Line 404. Insert consistently space between number and unit. One example where this not done is 0.45uM here. Check this throughout. – Noted and corrected.

Line 409. “When removal of the carboxyl terminal His-tag is needed” Stick to past time. – Noted and corrected, please see line 615.

Line 414. What buffer was used for the SEC purification? Is the column perhaps from Cytiva? – Buffer and column information was added, please see line 621.

Line 420. Mutation to Ala? – Noted and corrected, please see line 627.

Line 433. Screened for crystallization. – Noted and corrected, please see line 641.

Line 438. Reservoir condition supplemented with 20% v/v glycerol? – Noted and corrected, please see line 646.

Line 445. Data revealed an anomalous signal extending to 2 Å. How was this evaluated? Since the diffraction limit is also 2 Å, it is unlikely that the anomalous difference is significant at 2 Å also. Such a signal is weak and will normally not be significant in the last 0.5 Å resolution shell of such 2 Å data. Not that it matters for experimental phasing! It would also strengthen the manuscript if statistics for the experimental phasing in PHENIX was included in the manuscript. – Noted and the anomalous signal measurability has been corrected (line 655). The statistics for the experimental phasing have also been included in the method section, please see line 650-662.

Line 479. Where is the Titan microscope located? It is mentioned in the acknowledgements, but state it here also. – Noted and provided the location of the Titan Krios microscope in line 694.

Line 501. Can the difference in protomer orientation between the two 3D classes be quantitated? Are the two protomers differing by a simple small rotation or are there also internal differences within the protomers. RMSD values between protomers from the two different 3D classes could be given as a measure here. – Noted, see line 257-261 and S4D Fig. Since the resolution of the dimer maps were quite low for us to confidently build the models, we decided to only dock the AlphaFold2 structures into these maps to get a sense of how the protomer interact.

Line 568. Purified bovine FI? – Noted and edited, please see line 793.

Line 595. Correct "35 to 95oC" – Noted and corrected, please see line 818-819.

Line 613. "Supplementary Table 2" this must be extended table 2? – Noted and corrected, please see line 824 (now Table S7).

Line 640. This is Extended data 2, not 4. – Noted and corrected, please see line 841 (now Table S7).

Ext fig 2B. Very low resolution, export high-res from Excel – Noted and modified, please see updated figure S2B.

Legend fig 1. "Membrane was stained with ponceau S to show the amount of sample loaded per lane and detect cell lysis". It is not the membrane that is stained, it must be the proteins from the membrane – Noted and corrected, please see updated figure legend.

Figure 3. More close-up views to document the quality of the map would be beneficial. In b, the mesh thickness should be decreased or a transparent surface used instead for the

map. Also provide the contour level everywhere a map is presented. The local resolution map in the Extended figure could be moved here. Panel C, outline the interface footprint on molecules in the right panel. Panel E, how were these residues selected? From the structure only? – Noted and modified the figures. The reason for selecting these specific residues was also added in line 361-363.

Are there any conformational changes between the unbound crystal structure and PmSLP-1 in the complex with FI? – Noted, please see line 272-277 for added discussion.

Ext data table 1. How was the table generated? Phenix.table_one? As mentioned above, please include statistics from the experimental phasing on the anomalous data. – Noted and provided additional information on phasing in the method section. Please see line 650-662.

Ext data fig 1. The rmsd values given are not that meaningful. Provide the rmsd for the two domains separately and only for the common secondary structure elements. – Noted and modified, please see updated figure S1.

Ext data fig 4. The figure is so large that it should be split in two. Panel B should come early since it presents the sample rather than in the end – Noted and modified, please see updated figures S4 and S5.

Ext table 3. Coot is not used for refinement, only for rebuilding. Again, what program was used to generate the table? phenix.validation_cryoem? – Coot has been removed as a refinement package from Table S4, and the software used to generate the model statistics has been added to the method section (line 659-662).

Ext fig 5. Panel A. Give a reference to PISA , provide the weblink if the EBI server was used. Explain the meaning of P value from PISA and perhaps discuss whether it is a problem that the P value for the complex is close to 0.5. - We have added the meaning of P-value as supplementary info in S7A Fig. We don't think there is an issue with the P-value, because while the P-value of PmSLP-1:FI is close to 0.5, it is still within the range of P-values for a specific interaction (according to the PISA documentation).

Panel B. The C3b docking is made under the assumption that C3b conformation required for PmSLP-1:FI to cleave is the same as that required for FH:FI cleavage. This is not necessarily true. There are many structures of C3b in the PDB, and the location of the CUB domain relative to the MG-ring in a different known structure may reduce or remove the clash observed here for PmSLP-1 and C3b-MG6. Authors may want to compare with other known C3b containing structures. Also, pMSLP-1 bound FI may not require the CTC contact observed in the FI:FIH.C3b complex. – Thank you for the suggestion. We used AlphaFold3 to predict the structure of the ternary complex, which eliminated the clash between PmSLP-1 and C3b. However, AF3 predicted that part of the handle domain of PmSLP-1 formed helices instead of beta-strands. Therefore, we combined the AF3 model with our cryo-EM structure of PmSLP-1:FI to generate a new model for the PmSLP-1:FI:C3b complex. Please line 317-321 and the updated figure S8.

Ext data fig 6. Panel C. why are the crosslinks mapped onto AF predicted structure. Authors have an experimental structure, that should be trustworthy at the resolution the cryo-EM map. Or is there a specific reason? – The crosslinks have been re-mapped onto the cryo-EM structure of factor I and the measured distance have been updated accordingly (please see updated S9C Fig).

Reviewed by Gregers R Andersen. Thank you for the thorough review, Gregers.

Reviewer #2:
Minor Points:

1/ Fig. 2, legend: The word "condition" should be pluralized to "conditions" when describing preparation of samples for SDS-PAGE. Additionally, the legend inset of Fig 2d refers to a construct PmSLP-1^95, while the legend text refers to a construct PmSLP-1^94; please revise this accordingly. – Noted and corrected, please see the updated Figure 2 legend.

2/ Fig. 4, panels a, b: There does not seem to be a label, arrow, or text indicating the presence of the PmSLP-1 band in these SDS-PAGE images. This should be added to make this experiment easier to interpret for the readers. – We appreciate the suggestion for Fig 5 (previously Fig 4). However, the total amount of PmSLP-1 per reaction was less than 20 ng, which was too low for detection with Coomassie staining.

3/ Fig. EXD 6, legend: It seems the article "the" is missing from the title prior to "PmSLP-1:FI complex". Also, in the last sentence of this legend, the word "occurrence" should be pluralized to "occurrences". – Noted and corrected, please see the updated legend for S9 Fig.

4/ Abstract, line 13: The word "brutal" seems a bit excessive for the Abstract of a scientific manuscript. It might be worth removing this. – Noted and removed, please see updated abstract.

5/ Introduction, line 54: The authors suggest that 'cleaving of C3b and C4b into their inactive form….brings [all activity of] the complement cascade to a halt'. I think this is overstated, because its likely that there are many molecules of C3b and C4b generated upon complement activation and the word "halt" implies a sort of immediacy that's not likely seen when these natural regulatory mechanisms are in play. This problem can be eliminated by using a different word like "inhibited" or "diminished", etc. – Noted and replaced "halt" with "inhibit", please see line 69.

6/ Results, line 139: The article "the" is missing prior to the word "PmSLP-1 sample". – Noted and corrected, please see line 184.

7/ Results, line 154: Instead of using a generic word like "tight", it would be better if the authors used a more scientifically informative description like "a nanomolar-affinity" or "a

high-affinity” instead. – Noted and replaced “tight” with “high-affinity”, please see line 217.

8/ Results, lines 192-194: The authors write that “hydrophobic interactions also play a role in stabilizing the complex”. This is not necessary, as it is typical for protein-protein interactions – in fact, it would be very unusual if hydrophobic interactions weren’t involved to some degree. I recommend removing this sentence. – Noted, we elaborated on the analysis of the protein-protein interface, please see line 280-289.

Reviewer #3: General minor comment:
Figures 3 and 4 are multipaneled and spill over 2 pages, making it difficult to follow. May be a better idea to split them into several figures. – Noted and corrected, please see updated figures 3 and 4.

The crystal structure presented lacks 94 N-terminal residues. It would be helpful to provide the logic of the introduction of the “surface entropy reducing mutations”, or a general overview of the organization of the full-length molecule of PmSLP-1. – Noted and modified to provide more information, please see line 139-145.

The binding of the PmSLP-1 and FI is surprisingly tight, with a nanomolar affinity. The assessment of the docking interface is very diligent, and doesn’t simply rely on the cryo-EM structure or docking simulations, but a number of site-directed mutants are introduced and tested, which is very commendable. It would perhaps be helpful to show the putative interaction partners between E240 and respective FI residues, as the current Fig 3 isn’t very clear and E240 seems buried inside PmSLP-1. – Noted and modified, please see updated Figure 4B. The residue E240 of PmSLP-1 forms a salt bridge with R455 on factor I, which is shown in Fig. 3B, bottom right panel labelled “Site 2.”

Extended Data Fig 2b – the rendering of the table of the identity matrix is badly compressed and shows artifacts – please ensure that is rectified in the final version. – Noted and modified, please see updated figure S2B.

Extended Data Fig 3a/b – could hydrodynamic radius be calculated and used instead of the retention volume, which is column specific? – We have not calibrated this column with the MW standards. Our objective is to separate the monomers from the heterodimeric complex, so reporting the elution volume seems appropriate for this.

Extended Data table 4 – the list of plasmids should perhaps be a bit more detailed in specifying the exact length of the constructs, any linker regions and the cloning sites used. A UniprotKB (or other curated database) ID or the full sequence of the protein should also be provided, as it is for the majority of the other proteins discussed in the text, so one shouldn’t be forced to go via the PDB database to find it. – Noted and modified, please see updated list of plasmids and constructs in Tables S7 and S8.

Methods: please convert rpms to g-forces where applicable. Also, in some cases e.g. line 592 – formatting of the centigrade is missing. – All rpms have been converted to g-forces and the formatting for the centigrade has been corrected.
